# Diagnosing ASD in Children Aged 6–18: Gender Differences and the Diagnostic Process

**DOI:** 10.3390/jcm15020803

**Published:** 2026-01-19

**Authors:** Shahar Gindi, Hagit Nagar-Shimoni, Efrat Zilbershot Fink, Asi Fares, Noy Oppenheim, Yael Leitner

**Affiliations:** 1 Levinsky-Wingate Academic Center, Netanya 4290200, Israel; hagitna@tlvmc.gov.il; 2 Marot Autism Center, Child Development Institute, Dana-Dwek Children’s Hospital, Tel Aviv Sourasky Medical Center, Tel Aviv 6492403, Israel; efratfi@clalit.org.il (E.Z.F.); assifares@gmail.com (A.F.);; 3 Faculty of Medicine, Tel Aviv University, Tel Aviv 6997801, Israel

**Keywords:** gender differences, parental concerns, diagnostic tools, retrospective study, social interaction, language difficulties, late diagnosis neurodiversity, masking, social attribution test

## Abstract

**Background/Objectives**: Diagnosing ASD becomes more difficult with age, especially in girls. This study explores developmental factors and diagnostic tools that affect ASD diagnoses after age six. The study also integrates the neurodiversity paradigm to evaluate how diagnostic tools like the ADOS-2 and Social Attribution Test (SAT) capture the heterogeneous presentation of ASD across genders. **Methods**: This retrospective study analyzed data from 91 children (73 boys, 18 girls) assessed for ASD between ages 6–18. Multivariate Generalized Linear Models (GLMs) were employed to identify independent predictors of diagnosis, controlling for age, gender, and language difficulties. **Results**: Notable gender differences emerged: boys showed more atypical development and restricted interests, while girls showed higher sensory sensitivity. Multivariate analysis confirmed that Social Affect (SA), age of initial concern, and the absence of structural language difficulties significantly impacted diagnosis likelihood. **Conclusions**: This study emphasizes the need for gender-sensitive criteria and implicit measures like the SAT to identify “masking” phenotypes. It emphasizes current tool limitations, the risk of diagnostic overshadowing, and the importance of longitudinal studies with comprehensive assessments to better capture ASD diversity, especially in social and language skills.

## 1. Introduction

Autism spectrum disorder (ASD) is a neurodevelopmental condition characterized by challenges in social communication and the presence of restrictive/repetitive behaviors [1]. The CDC’s ADDM Network estimates 1 in 36 children has an ASD diagnosis [2]. The male-to-female ratio (MFR) in children is 3.67 overall, but varies with comorbidity: MFR is 3.94 in children with typical intellectual functioning, drops to 2.62 in those with ID, and rises to 4.26 in ASD children with ADHD [3].

The evolution of diagnostic practices for ASD may transition in the coming years toward a more integrated, multimodal approach. Researchers are increasingly exploring the integration of genetic evaluations, telemedicine, and digital technologies [4]. Furthermore, machine-learning-based analyses and digital diagnostic tools show promise in identifying subtle behavioral patterns, yet these innovations require larger, more robust studies to ensure reliability. At present diagnosis relies on developmental history and clinical observation, a process heavily influenced by the expertise of the clinician and the absence of established diagnostic biomarkers.

ASD diagnostic criteria focus on the presence of specific behaviors or impairments within two domains: (1) social-communication/interaction impairments, and (2) restricted, repetitive patterns of behavior (RRB), interests, or activities [1]. The ASD Diagnostic Observation Schedule, Second Edition (ADOS-2) is a key diagnostic tool [5]. ADOS assesses both Social Affect (SA) and RRB [6]. A standardized version, the Calibrated Severity Scores (CSS), provides a total score for ASD symptom severity that controls for background factors like age and language skills [7]. CSS allows severity comparison across individuals with ASD despite differing developmental levels [8].

While the ADOS-CSS is advantageous, the symptoms contributing to an individual’s score can vary; a high score might stem from social-communication difficulties alone or a moderate combination of social-communication and RRBs. Social-communication challenges often involve the absence of typical behaviors (e.g., lack of gestures or eye contact), whereas RRBs are atypical actions (e.g., hand flapping). Assessing RRBs is difficult due to their context-specific nature, limiting the ADOS in time and context regarding RRBs [7]. Despite this, both social-communication and repetitive behaviors measured by the ADOS reliably predict an ASD diagnosis [9]. The SA domain is considered more reliable than the RRB domain in the ADOS-2, with SA items showing greater variability [8]. Consequently, a high SA score can predict autism-related social-communication difficulties. Furthermore, research highlights the accuracy of the SA domain by finding significant differences between ASD and non-ASD patients’ SA scores, with ASD patients scoring higher [10].

Despite the many strengths of the SA domain, alone it lacks specificity, which means a higher rate of false positive diagnosis of ASD. Therefore, the SA domain must be included with the RRB domain for a better specificity and diagnosis [11]. Additionally, if children disregard or respond unfavorably to an examiner’s efforts to engage them in a game during the ADOS, they may be assigned a non-zero score to their social response quality. Impulsive or hyperactive behavior might disrupt activities like storytelling during the ADOS, leading to a non-zero score for the overall rapport quality. These issues harm the specificity of the SA domain leading to increased false positive cases in diagnosing ASD [8].

Parental concerns and recognition of symptoms are crucial in the pathway to an ASD diagnosis. Studies have highlighted the importance of parental concerns and their relation to the age at diagnosis, with evidence suggesting that the severity of ASD relates to earlier parent concern [12]. The age of ASD diagnosis also correlates with the child’s age when parents first voiced concerns [13].

Language delay leads to an earlier age of concern, resulting in an earlier age of diagnosis [14]. Conversely, when parental concerns relate to cognitive developmental delay, it often leads to a later diagnosis for children whose mothers have low education or low family income. Mothers with less education face greater difficulty accessing healthcare services, including diagnosis and early intervention. When children show cognitive or adaptive developmental delays, parents with lower educational backgrounds may delay seeking healthcare assistance compared to those with higher educational attainment [15].

The presentation of symptoms in some children evolves, meaning they may only meet diagnostic criteria later. Clinicians sometimes overlook ASD features during early evaluation, leading to a late diagnosis as social needs and difficulties become more challenging [16]. A study using the SPARK cohort identified two distinct subgroups among individuals with delayed autism diagnoses (after age 6) [17]. One subgroup had lower support needs and fewer comorbidities; the second had higher support needs and more co-occurring conditions, indicative of diagnostic overshadowing.

The challenges in diagnosing ASD are exacerbated in girls. Research consistently shows that girls, particularly those without cognitive impairment, are less likely to receive a timely diagnosis than boys [18]. This disparity may result from subtle symptom presentation in females, often causing delayed recognition and intervention. A longitudinal study revealed distinct developmental trajectories of autistic social traits in males and females, with a notable increase in autistic traits among girls during adolescence [19]. Clinical observations suggest females with ASD may superficially display better social and emotional skills than males, potentially masking other diagnostic features [20].

A growing body of evidence suggests ASD may overlap with specific language impairment (SLI) in social and communicative deficits. The overlap between ASD and SLI is based on the structural and social abnormalities seen in individuals with SLI and their similarity to the pragmatic impairment and language abnormalities observed in individuals with ASD [21]. Research on language deficits in children with developmental disorders like developmental language disorder (DLD) and ASD indicates that children with DLD have difficulties with language structure [22]. Conversely, children with ASD typically struggle with pragmatics, defined as using language correctly in context [23]. The pragmatic difficulties in ASD led researchers to hypothesize that ASD is also accompanied by structural language difficulties [24]. Thus, a potential overlap exists between ASD and DLD, where language difficulties manifest differently but stem from a common source.

The current improvements to the most widely used ASD diagnosis tool, the ADOS-2, are helpful; however, they provide less optimal outcomes in complex cases with differential diagnostic issues [25]. Specifically, these improvements may lead to elevated numbers of false positive diagnoses (up to 29%) in children with other psychological conditions and disorders, and specificity issues remain a great concern [11,26].

A child’s presentation of ASD symptoms is sometimes subtle or complicated by co-existing health concerns, resulting in a complex medical or psychosocial history [27,28]. These factors challenge diagnostic determinations. In such complex cases, when golden standard tests and consultation among professionals are insufficient for an accurate diagnosis, some clinicians utilize a multidisciplinary team (MDT) approach. This involves a collaborative team of health care professionals conducting diagnostic assessments. Interdisciplinary teams work cohesively and in an integrated manner. Multidisciplinary teams, conversely, operate independently but share information, potentially achieving a diagnostic consensus. Though this process can be lengthy, potentially delaying early diagnosis, it is effective in gaining a more accurate diagnosis in complex cases [29].

The existing ASD diagnostic tools face multiple limitations, including clinician subjectivity, age-specific challenges, cultural insensitivity, gender bias, complications from coexisting conditions, limited sensitivity/specificity, and potential caregiver reporting bias [30]. Therefore, to properly assess subtle variations in social behavior and communication, additional thorough clinical observation and assessment are needed to complement structured modules [31].

This study aims to examine the unique developmental parameters and diagnostic tool characteristics that influence ASD identification in youth aged 6–18. By employing multivariate modeling and integrating a broad range of psychosocial perspectives, we seek to inform more equitable and efficient diagnostic procedures that appropriately address the diversity of the autism spectrum.

## 2. Materials and Methods

### 2.1. Context

As an ASD center within a public hospital, we provide comprehensive, multidisciplinary ASD diagnostic evaluations for children aged 6–18 across Israel. Referrals are commonly initiated by professionals like neurologists, psychiatrists, and psychologists due to observed challenges with social communication and, at times, social isolation. This research was conducted per the Declaration of Helsinki and the Ethical Principles of Psychologists and Code of Conduct. The hospital’s Institutional Review Board (#0502-21) approved the research. Consent was not required as this was a retrospective study examining medical records. To ensure the privacy of all participants, the data were fully anonymized prior to analysis. All personally identifiable information, such as names and identification numbers, was removed from the dataset, and replaced with unique coded identifiers to maintain confidentiality and strictly protect the anonymity of the medical records throughout the study.

### 2.2. Measures

Our assessments employ a comprehensive, team-based approach, integrating multiple data sources for accurate evaluations. These sources include standardized teacher and parent questionnaires, an in-depth parent interview for developmental history and current concerns, and a structured interview meticulously aligned with the DSM-5 diagnostic criteria. Each evaluation is conducted by experienced physicians and psychologists who bring the results of their evaluations to clinical case conferences, where they are discussed in consultations to reach the final conclusions and finalize the diagnosis. The clinicians utilize the following standardized tools:

The Autism Diagnostic Observation Schedule—Second Edition (ADOS-2): This is a semi-structured, standardized assessment of communication, social interaction, play, and imaginative use of materials. It provides a systematic way to observe behaviors relevant to the diagnosis of ASD across different age groups and developmental levels. Our analysis focuses on key summary scores including the SA Total, RRB Total, and the Total (SA + RRB) score to quantify the severity of ASD-related symptoms. The study used an interrater agreement protocol whereby clinicians would bring the ADOS results to weekly clinical case conferences. Thus, while there are no statistical interrater agreement numbers to present as such, the ADOS scoring was scrutinized by colleagues.

DSM-5 Diagnostic Criteria for ASD: We adhere strictly to the diagnostic criteria outlined in the Diagnostic and Statistical Manual of Mental Disorders, 5th Edition (DSM-5) for ASD. This involves a careful evaluation of the individual’s presentation against the specific criteria related to deficits in social communication and social interaction (A1-3), as well as the presence of restricted, repetitive patterns of behavior, interests, or activities (B1-4).

The Social Attribution Test (SAT): The SAT is a nonverbal social cognitive procedure developed to reduce the confounding potential of verbal mediation by minimizing explicit instructions. The SAT requires participants to view a 50 s clip showing geometric figures moving contingently [32,33]. We utilize the SAT Salience index, which measures participants’ social interpretation by counting the social elements spontaneously mentioned; a higher score reflects better social ability. Previous research demonstrated this index’s effectiveness in identifying social cognitive deficits associated with ASD in girls [31]. Because the Salience index relies on spontaneous narrative rather than explicit prompts, it helps clinicians expose social difficulties that girls often mask through the camouflage effect. While girls may outperform boys when given a specific clue, as seen in the SAT Person index, the lack of significant differences in spontaneous indices suggests that the SAT is a useful tool for circumventing camouflaging behaviors.

### 2.3. Participants

The study used data from 91 children whose age at first concern was 1 to 15 years (M = 5.14, SD = 2.90). The sample included 73 boys (80.2%) and 18 girls (19.8%), and their age upon clinic arrival ranged from 6 to 18 years (M = 11.67, SD = 3.40). The male-to-female ratio (MFR) is consistent with the literature [3]. Although girls were slightly younger than boys at the time of first concern (M = 4.65, SD = 2.68 vs. M = 5.24, SD = 2.96), this difference was insignificant (t = 0.706, *p* = 0.241), as was the difference in age at clinic visit (M = 12.56, SD = 3.57 vs. M = 11.45, SD = 3.35; t = 1.237, *p* = 0.219). The mean time from first concern to diagnostic arrival was 6.43 years (SD = 3.58, range: 0.00–15.00). Only 84 children provided full data, so subsequent analyses used this sub-sample. The next section details the sample characteristics.

### 2.4. Design

The empirical analyses had two parts. The preliminary part used the *X*^2^ test to compare frequencies of major characteristics between boys and girls, and the two-independent t-test for gender comparisons of continuous scales (age of first concern, relevant score measures). The second part involved in-depth analyses using the generalized linear model (GLM) framework with robust standard errors to explain the children’s diagnosis. The outcome was a binary diagnosis (yes = 1), complemented by a division between physicians and psychologists to assess predicted probabilities for a child to affiliate with one of three options: without, with, and referral for additional assessment. All options were predictors of the final binary diagnosis.

## 3. Results

Table 1 provides frequencies and descriptive statistics for main sample background characteristics and evaluations. A gender comparison (boys versus girls) was performed across all categorical and continuous variables (Chi-Square, two-independent sample t tests, respectively) in the tables, which followed previous studies . Differences were found in rates of atypical development, higher rates among boys than among girls (80.3%, 56.3%, respectively, *p* = 0.04), and limited interest (B3), which were significantly higher among boys than among girls (94.4% versus 54.5%; *p* < 0.001). Sensitivity (B4) was higher among girls (100.0% versus 72.2%; *p* = 0.049). Physicians’ recommendations varied significantly between boys and girls. Girls received recommendations for additional observation 53.8% of the time and were diagnosed with no autism 30.8% of the time. In contrast, boys were recommended as follows: no autism 30.8%, autism 30.8%, and further assessment 26.9% (*p* = 0.004). The use of SSRIs was significantly higher in girls compared to boys (46.7% versus 6.3%; *p* < 0.001). Other tested indicators did not indicate gender differences. Comorbidity rates were above 90 percent, while ADHD was found in 64.4% of children surveyed. Notably, the low rates of girls in this sample raise concerns about analytical power, yet this unbalanced rate represents the unbalanced rate in the population [3].

Table 1 continues this comparison (two-independent sample *t*-test) and shows continuous research indicators. The table shows that the mean age at which concerns were raised was slightly above five years of age (SD = 2.90). Among the behavioral indicators on the list, only the Salience index of the SAT was found to be higher among boys in comparison to girls (*p* = 0.035). Thus, the main conclusion was to continue with the full sample without gender control, except for the unique models that included B3 and B4, as well as the Salience index, as indicators for the final diagnosis. For the latter it meant that gender effect should be included as main effect and probably in interaction with the confounded indicators. Although the rate of diagnosed girls was higher than among boys (61.1% versus 50.0%), this difference was found to be insignificant (*p* = 0.399) but indicated that only half of applying cases were eventually diagnosed. Table 1 shows the ADOS scores in two forms: as continuous (Table 1: M = 1.90, SD = 0.94), and as an ordinal variable (not ASD (1): 49.3%; ASD (2): 11.3%; autism (3): 38.3%). For analytical purposes, we used ADOS as an independent continuous variable. Table 1 shows continuous indicators followed by a gender comparative t-test. Across all these indicators, the Salience index of the SAT was found to differ, such that the mean for boys was higher (Boys: M = 0.37, SD = 0.16; Girls: M = 0.30, SD = 0.14; t = 1.84, *p* = 0.035). A follow-up analysis was performed on the Salience index to test whether diagnosed and undiagnosed vary by gender and vice versa. In other words, to what extent were diagnosed girls and boys similar? A two-way ANOVA model was performed, which resulted in an insignificant gender by diagnosis interaction. This means that although girls were found to be lower on the Salience index, on average, in comparison to boys, we could not say that this difference was inconsistent with respect to the diagnosis status. Finally, the physicians’ and psychologists’ diagnoses across boys and girls were found to be consistent, and no gender differences in these diagnoses were found.

**Figure 1:**
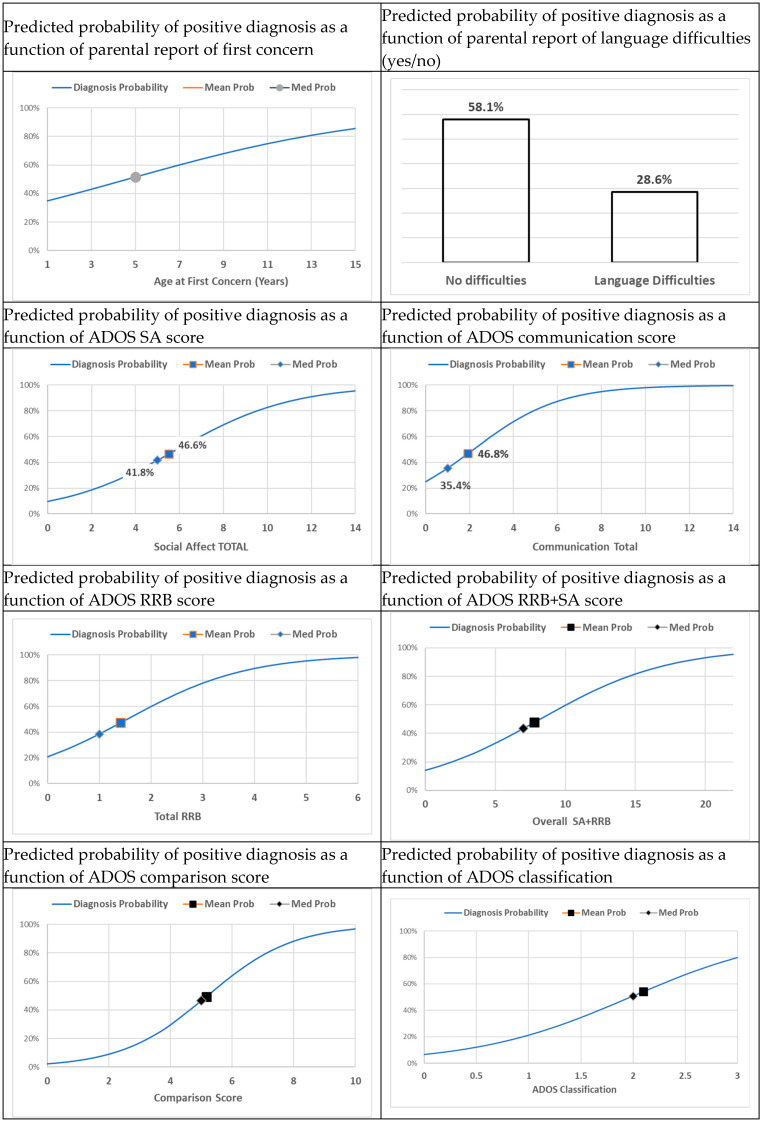
Predicted probability of positive diagnosis as a function of various background indicators.

**Figure 2:**
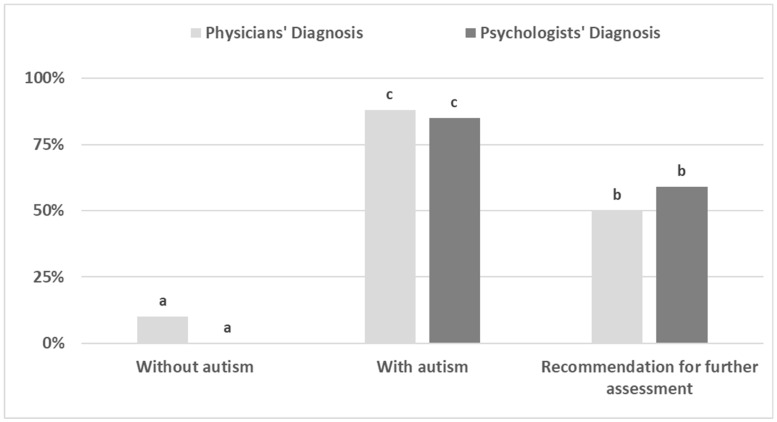
Probability of positive diagnosis with respect to categorical assessments by psychologists and physicians. Note: Latin letters for predicted marginal mean ranking from the lowest “a” and upward.

For the purpose of this study, we ran a list of independent models to assess each score effect on the probability of being diagnosed. Table 2 shows GLM results, in which the outcome was the final diagnosis, a binary outcome in which diagnoses were coded one. Each row represents one independent model’s results. For instance, the total SA score was found to be associated with an increasing probability of positive diagnosis (b = 0.37, *p* = 0.001, ODDs = 1.44). This means that the probability of positive diagnosis increased by 1.44 times more than the probability of negative diagnosis with respect to a unit change in the total value of SA, where a unit change means that SA changed by one unit on a scale from one to six. Note that the Benjamini–Hochberg (BH) correction was applied to adjust the *p*-value for multiple tests [34]. To illustrate this increase and other effects on the probability of becoming diagnosed, Figure 1 depicts this probability as a function of the explanatory variable. The horizontal axis shows values of the explanatory variable, and the vertical axis shows probabilities (percent). Thus, these probabilities are a function of the explanatory variable, and a unit change in the former makes a probability change in the latter, but this change is non-linear. We also see that age when concerns first appeared was associated with higher probability of positive diagnosis (b = 0.17, *p* = 0.055, ODDs = 1.19). That is, if concerns appeared at an older age, the chances of being diagnosed with autism were higher than if these concerns appeared at a younger age. Language difficulties indicated a lower probability of positive diagnosis (b = −1.24, *p* = 0.054, ODDs = 0.29), which suggested that earlier concerns were in fact related to the language difficulties. Higher SA scores were positively associated with the probability of positive diagnosis (b = 0.51, *p* = 0.015, ODDs = 1.66). Other indicators were positively associated with the probability of positive diagnosis, e.g., RRB, Overall SARRB, comparison score, and ADOS classification. These significant effects on the probability of eventual diagnosis are illustrated in Figure 1. For continuous indicators of this probability a full cumulative probability line in response to values of the independent (explanatory) factors is illustrated, whereas dichotomous indicators were presented as bars, e.g., language difficulties (see Figure 2).

Table 3 shows associations between physicians’ and psychologists’ evaluations (independently) and the predicted probability of positive diagnosis. In other words, what was the probability of positive diagnosis if the physician (or the psychologist) evaluated the case as one of the four alternative categories, namely, the association between each possibility that physicians and psychologists used and the probability of those who were evaluated as such to eventually be positively diagnosed? Among psychologists, none of the cases initially evaluated as without autism were predicted as finally diagnosed with autism (predicted probability of 0.00%), while all provisional cases were predicted as positive diagnosis among the psychologists’ evaluations. If psychologists diagnosed the cases as a case with autism, the predicted probability of final diagnosis was 85 percent. Overall, the preliminary psychologists’ evaluations were found to be insignificant in predicting the final decision outcome. Among physicians, of those who were evaluated with autism (eventually 88%) were diagnosed, and ten percent, who were not diagnosed by psychologists, were eventually diagnosed as with autism. The physicians’ evaluations were found to be associated with the final decision (Wals’s *X*^2^ = 15.84, *p* < 0.001). The outcome probabilities are shown in Figure 2, in which the Latin letters represent ranking from the lowest probability (a) and onwards.

For illustration, Figure 1 presents the predicted probability to be diagnosed with respect to changing values of independent indicators. For continuous indicators, we show the predicted cumulative probabilities, and for binary indicators we show the predicted probabilities in two bars. Interestingly, in response to language difficulties, the predicted probability of being diagnosed was smaller than that of cases without difficulties. This counterintuitive outcome can be explained by the fact that language difficulties brought the child for diagnosis, yet these difficulties were not comorbid with autism. In other words, language difficulties were not an indicator of autism. Another indicator was ADOS. In the figure, the predicted probability of being diagnosed increased with respect to increasing levels of ADOS, where ADOS is a categorical scale (1 to 3) from not being on the spectrum to autism.

## 4. Discussion

This study reveals significant gender differences in ASD presentation and diagnosis, underscoring the need for refined diagnostic approaches. Higher scores among boys than girls on autism-related traits (Table 1) align with established literature on male predominance [20]. However, the lack of a significant interaction effect between gender and diagnosis suggests this difference is not solely diagnostic bias, supporting the notion that higher average scores in boys reflect genuine differences in trait expression. This resonates with research highlighting challenges girls face in timely diagnosis due to subtle symptoms [18,19]. The emphasis on social communication difficulties aligns with established literature prioritizing the SA domain on the ADOS in ASD diagnosis [7,8]. While earlier parental concern correlates with earlier diagnosis, the impact of language difficulties suggests a complex relationship; early language challenges might hinder timely ASD identification [14]. The substantial contribution of the RRB domain to diagnosis likelihood further emphasizes considering the full spectrum of ASD symptoms [5,7].

The inverse relationship between recommendation for further evaluation and the likelihood of a positive ASD diagnosis warrants further investigation. This additional evaluation step led to a lower overall probability of diagnosis compared to the initial expression of parental concerns. This may be because the decision implies a degree of uncertainty or doubt among clinicians regarding the initial assessment findings. This uncertainty may stem from various factors, including the subjectivity inherent in current diagnostic tools, the presence of co-occurring conditions that complicate diagnosis [9,31], and the age-specific challenges in accurately assessing ASD [30].

The study’s focus on developmental parameters and diagnostic tools beyond early childhood, especially concerning the difficulties in identifying ASD in females, highlights the complexity of the diagnostic process across the lifespan. The observation that later-emerging developmental concerns correlate with a higher likelihood of ASD diagnosis within the 6–18 age range presents a noteworthy departure from what is often observed in younger children [14]. Typically, diagnostic indicators in children below age six tend to focus heavily on early developmental milestones, particularly in the realms of language and social interaction. A delayed onset of such concerns within the older age bracket may signal a different manifestation of autism, perhaps one characterized by more subtle social communication differences that become more apparent as social expectations increase with age.

Furthermore, the finding that language difficulties appeared to reduce the probability of a positive diagnosis within this older cohort prompts intriguing questions. In younger children, language delays are frequently a key indicator of potential ASD [21]. The opposite trend observed in the 6–18 age group may reflect a survivorship bias (or selection bias) within the diagnostic pipeline. In early childhood, language delay is a primary “red flag” that triggers immediate clinical referral, meaning children with both ASD and language difficulties are often identified and diagnosed well before age six. Consequently, these children are effectively filtered out of the undiagnosed pool by the time they reach school age. Those diagnosed later in development (ages 6–18) are disproportionately likely to have intact or superior linguistic skills, as their lack of early language delay allowed their social communication differences to remain hidden until the social demands of adolescence became more complex. Thus, in an older cohort, the presence of language difficulty may appear to “reduce” the probability of a new ASD diagnosis simply because those who fit that profile were already caught by the system years prior. This suggests the need for assessment tools and diagnostic criteria that are sensitive to the evolving presentation of ASD across different developmental stages and age groups, moving beyond a singular focus on early language skills.

The diagnostic process for ASD is revealed to be subject to considerable inter-professional variability, as highlighted by the comparison of physician and psychologist evaluations (Table 3). This variability in diagnostic outcomes underscores the need for a more thorough examination of factors influencing clinical judgment. Differences may arise from variations in applying diagnostic criteria [30], methodological approaches [15], or even inherent biases among professionals. The study reveals noteworthy discrepancies in diagnostic outcomes, potentially stemming from fundamental differences in the initial assessment strategies employed by physicians and psychologists. This disparity, reflected in the varying rates of false positives, suggests that a more in-depth analysis of the assessment processes themselves is warranted. While both groups aimed to evaluate the presence or absence of ASD, the results hint at differing approaches in terms of the scope of indicators considered, the level of detail in their clinical evaluations, and perhaps even in the application of diagnostic criteria. For example, psychologists may have incorporated a broader range of assessment tools, and spending more time with the children may have given them more diagnostic observation materials [9,30]. This comprehensive methodology may have contributed to the significantly lower rate of false positives observed in the psychologists’ evaluations. Furthermore, psychologists often prioritize behavioral observations and developmental histories, whereas physicians may rely more heavily on immediate clinical presentations or standardized medical screenings.

The diagnostic process must serve as a critical catalyst for the implementation of evidence-based psychosocial interventions tailored to the specific social-cognitive profile of the individual. Gosling et al. [35] identified several pathways with established efficacy for school-aged children and adolescents. Specifically, the implementation of Social Skills Groups (SSG) is supported by suggestive evidence for improving social-communication deficits and overall ASD symptom severity in this age group [36]. Furthermore, interventions such as Cognitive Behavioral Therapy (CBT) are vital for high-functioning teens, as they focus on developing coping skills that enable individuals to modify the maladaptive thoughts and emotions that frequently contribute to social-affective challenges [37]. Beyond traditional behavioral frameworks, emerging social catalysts such as theater-based interventions [38], dog-assisted therapy (DAT) [39], and Interventions aimed at improving emotional intelligence [40] offer promising avenues for adolescents who struggle with reciprocity.

For adolescents referred after age six, clinical practice must move beyond male-normed behavioral checklists and integrate measures sensitive to subtle presentations. Our findings suggest that diagnostic accuracy is significantly enhanced by the inclusion of implicit social-cognitive measures, such as the Social Attribution Test (SAT), which can circumvent the verbal camouflage often employed by high-functioning individuals [32,33]. Clinicians should prioritize the assessment of social camouflaging using specialized metrics to identify individuals who maintain surface-level social competence despite significant underlying distress. Professional training programs must be updated to recognize the female ASD phenotype, emphasizing that restricted interests in girls are frequently age- and gender-congruent (e.g., animals or fiction), and promoting interprofessional education to reconcile the medical lens of physicians with the psychologists’ lens. This is urgent because the delay in diagnosis, averaging 6.43 years in our sample, has severe psychiatric and functional consequences, including an elevated risk for burnout due to the sustained effort of masking, and increased susceptibility to social victimization.

Future research utilizing longitudinal designs, examining diverse populations, and employing more refined clinical measures is crucial for advancing our understanding of ASD and for developing more accurate and efficient diagnostic procedures that appropriately address these complexities [4,17,29]. Ultimately, this research underscores the need for diagnostic criteria that better account for the heterogeneity of ASD presentations, particularly those impacting social interaction and language development.

### Study Limitations

This study’s findings should be interpreted in light of several limitations. The cross-sectional design, while offering a broad snapshot, prevents the examination of developmental trajectories over time. Additionally, the sample, while large, is not fully representative of the general population and is skewed towards high-functioning individuals, potentially limiting the generalizability of findings to lower-functioning individuals. The relatively small sample size of girls (18 compared to 73 boys) may also limit the power to detect significant sex differences. Finally, the retrospective nature of the data collection regarding the timing of parental concerns and age at diagnosis introduces recall bias and limits the precision of these measures.

## Figures and Tables

**Table 1:** Main research indicators—frequencies and descriptive statistics for continuous indicators by gender.

	Boys				Girls				All					*p*-Value
	Freq.		Pct.		Freq.		Pct.		Freq.		Pct.		χ^2^	
	71		80.2		18		19.8		91		100			
Diagnosis by DSM	36		50.0		11		61.1		47		52.2		0.71	0.399
Atypical Development	57		80.3		9		56.3		66		75.9		4.12 *	0.042
B1 Positive	21		58.3		5		45.5		26		55.3		0.56	0.452
B2 Positive	20		55.6		8		72.7		28		59.6		1.03	0.310
B3 limited interests	34		94.4		6		54.5		40		85.1		10.58 ***	<0.001
B4 hypo- or hyper-sensitivity	26		72.2		11		100		37		78.7		3.88 *	0.049
Comorbidities	64		90.1		18		100		82		92.1		1.93	0.165
ADHD	49		68.1		9		50.0		58		64.4		2.05	0.152
Anxiety	18		26.9		5		35.7		23		28.4		0.45	0.504
Learning disabilities	21		31.3		7		38.9		28		32.9		0.37	0.545
Language difficulties	12		19.4		3		20		15		19.5		0.003	0.955
Pharmacological interventions	29		43.3		10		62.5		39		47.0		1.92	0.166
Stimulants	24		36.9		5		33.3		29		36.3		0.07	0.794
Antipsychotics	10		15.2		4		28.6		14		17.5		1.44	0.230
SSRIs	4		6.3		7		46.7		11		13.9		16.56 ***	<0.001
ADOS													0.49	0.783
Not ASD	28		50.0		5		45.5		33		49.3			
ASD	6		10.7		2		18.2		8		11.9			
Autism	22		39.3		4		36.4		26		38.8			
Psychologists’ opinion													1.85	0.396
Without autism	22		37.3		3		21.4		25		34.2			
With autism	19		32.2		7		50.0		26		35.6			
Further assessment	18		30.5		4		28.6		22		30.1			
Physicians’ Opinion													3.40	0.182
Without autism	16		30.8		4		30.8		20		30.8			
With autism	16		30.8		1		7.7		17		26.2			
Further assessment	20		38.4		8		61.5		28		43.1			
	**Boys**				**Girls**				**All**					
	**Mean**	**SD**		**N**	**Mean**	**SD**		**N**	**Mean**	**SD**		**N**	**T**	***p***
Age at 1st concern	5.24	2.95		69	4.55	2.68		15	5.13	2.90		84	0.70	0.482
Age at visit	11.45	3.35		72	12.56	3.57		18	11.67	3.40		84	−1.24	0.219
Communication	1.90	1.89		39	2.20	0.84		5	1.93	1.80		44	−0.63	0.271
SA Total	5.63	4.22		40	4.60	3.13		5	5.51	4.09		45	0.52	0.302
RRB Total	1.51	1.57		39	0.60	1.34		5	1.41	1.56		44	1.24	0.222
Total (SA + RRB)	8.19	6.32		57	5.73	3.88		11	7.79	6.04		68	1.24	0.109
ADOS class.	1.89	0.95		56	1.91	0.94		11	1.90	0.94		67	0.05	0.959
Comparison	5.37	3.37		38	3.83	2.32		6	5.16	3.27		44	1.07	0.145
Salience index	0.37	0.16		73	0.30	0.14		18	0.36	0.16		91	1.84 *	0.035

*** *p* < 0.001, * *p* < 0.05.

**Table 2:** GLM analysis results for the probability of autism diagnosis.

	b	Se	EXPB	*p*	BH adj.	N
Age at 1st concerns	0.17	0.09	1.19	0.055		83
Age at visit to the clinic	0.17	0.09	1.19	0.055		83
Language difficulties	−1.24	0.65	0.29	0.054		76
SA Total	0.37	0.11	1.44	0.001	Sig.	44
Communication	0.51	0.21	1.66	0.015		43
RRB Total	0.87	0.31	2.39	0.004	Sig.	44
Total (SA + RRB)	0.22	0.06	1.25	<0.001	Sig.	67
Comparison score	0.72	0.20	2.06	<0.001	Sig.	43
Classification-ADOS	1.35	0.33	3.85	<0.001	Sig.	66
Salience index	1.12	1.37	3.06	0.414		90
Atypical Development	0.52	0.51	1.68	0.315		86
Comorbidities	−0.26	0.80	0.77	0.741		88
ADHD	0.57	0.45	1.77	0.201		89
Anxiety	0.23	0.50	1.26	0.648		80
Learning disabilities	0.00	0.46	1.00	1.00		84
Pharmacological interventions	0.26	0.44	1.29	0.559		83
Stimulants	0.39	0.47	1.47	0.409		80
Antipsychotics	0.17	0.59	1.18	0.779		80
SSRIs	0.50	0.67	1.65	0.456		79

**Table 3:** The effect of physicians’ and psychologists’ assessment on the final diagnosis.

	N	Actual %	WALD	Marginal Means	SE
Psychologists’ opinion			3.70		
Without autism	25	34.2		0.00	0.00
With autism	26	35.6		0.85	0.07
Recommendation for further assessment	41	28.4		0.38	0.11
Physician’s Opinion			15.84 ***		
Without autism	20	30.8		0.10 ^a^	0.07
With autism	17	26.2		0.88 ^c^	0.08
Recommendation for further assessment	28	43.0		0.50 ^b^	0.09

*** *p* < 0.001. Marginal means are predicted probabilities. Lowercase superscripts (a, b, c) indicate statistically significant differences between groups. Marginal means that do not share a common letter are significantly different from one another at the *p* < 0.05 level.

## Data Availability

The data presented in this study are available in the Mendeley Data repository at https://data.mendeley.com/datasets/43923hs7rv/1 (accessed on 4 January 2026).

## Abbreviations

The following abbreviations are used in this manuscript:

ASD	Autism Spectrum Disorder
ADOS	Autism Diagnostic Observation Schedule
